# Prognostic Value of Ratios of Inflammatory Markers in the Prognosis of Crimean–Congo Hemorrhagic Fever

**DOI:** 10.3390/tropicalmed10040099

**Published:** 2025-04-08

**Authors:** Mürşit Hasbek, Yasemin Çakır Kıymaz, Seyit Ali Büyüktuna, Hayrettin Yavuz

**Affiliations:** 1 Department of Medical Microbiology, Faculty of Medicine, Sivas Cumhuriyet University, Sivas 58000, Turkey; 2 Department of Infectious Diseases and Clinical Microbiology, Faculty of Medicine, Sivas Cumhuriyet University, Sivas 58000, Turkey; yasemincakirkiymaz@cumhuriyet.edu.tr (Y.Ç.K.); seyit@cumhuriyet.edu.tr (S.A.B.); 3 Department of Nephrology, Faculty of Medicine, Virginia University, Charlottesville, VA 22903, USA; eeu9ng@virginia.edu

**Keywords:** Crimean–Congo hemorrhagic fever, inflammations, mortality, prognosis

## Abstract

Crimean–Congo hemorrhagic fever (CCHF) is a tick-borne zoonotic disease, causing clinical presentations ranging from asymptomatic infection to fatal viral hemorrhagic fever. Throughout the course of CCHF, the levels of certain biomarkers, such as platelets (PLTs), white blood cells (WBCs), C-reactive protein (CRP), and interleukin-6 (IL-6), may vary, decreasing below or rising above normal limits. This study aimed to investigate the role of parameters such as WBC/PLT, WBC/IL-6, WBC/CRP, and WBC/D-dimer ratios in predicting disease prognosis in patients diagnosed with CCHF. The study population consisted of 60 CCHF patients and 30 controls. Statistically significant differences were observed in hemoglobin (HGB), PLT, WBC, activated partial thromboplastin time (aPTT), international normalized ratio (INR), fibrinogen, and d-dimer values between the patients and controls. Statistically significant differences were observed in WBC/aPTT, WBC/fibrinogen, WBC/D-dimer, and WBC/IL-6 values between the patient and control groups. WBC/INR and WBC/fibrinogen values were lower in fatal cases compared to survivors. WBC/D-dimer and WBC/IL-6 values, on the other hand, were higher in fatal cases compared to survivors. In patients requiring intensive care unit (ICU), WBC/PLT, WBC/INR, WBC/aPTT, and WBC/fibrinogen values were higher compared to those who did not. However, WBC/D-dimer and WBC/IL-6 values were lower in patients requiring ICU compared to those who did not.

## 1. Introduction

Crimean–Congo hemorrhagic fever (CCHF) is a tick-borne zoonotic disease with a wide geographical distribution, causing clinical presentations ranging from asymptomatic infection to fatal viral hemorrhagic fever [1,2]. The causative pathogen is the CCHF virus (CCHFV), which belongs to the *Orthonairovirus* genus of the *Nairoviridae* family in the *Bunyavirales* order, with cases frequently reported from Africa, the Middle East, Asia, and Southern and Eastern Europe [3]. The expanding habitat of *Hyalomma* genus ticks increases the spread of the disease [4]. There is no licensed vaccine or specific antiviral treatment option for CCHF [5]. Moreover, despite the annual reports of CCHF cases, the host and viral determinants involved in the pathogenesis of the disease have not yet been fully understood [6].

CCHF is a viral hemorrhagic disease characterized by thrombocytopenia, leukocytosis or leukopenia, coagulopathy, and systemic inflammatory response. Certain laboratory markers, including low platelet count (PLT), high leukocyte count (WBC), prolonged activated partial thromboplastin time (aPTT) and elevated D-dimer and interleukin-6 (IL-6) levels, have been found in prior research to be possible predictors of disease severity and mortality [7,8,9].

Recent evidence indicates that assessing the ratios of inflammatory and coagulation markers, rather than individual markers alone, may offer more valuable prognostic insights by reflecting dynamic interactions across multiple physiological pathways. Among these ratios, the platelet-to-lymphocyte ratio (PLR) and neutrophil-to-lymphocyte ratio (NLR) have been widely studied as prognostic markers in various inflammatory and infectious diseases, including sepsis, pneumonia, and viral hemorrhagic fevers [10,11,12,13]. In CCHF, platelet levels typically decrease due to viral-induced endothelial damage and disseminated intravascular coagulation (DIC), while leukocyte response varies depending on the stage of infection and immune activation [2,14]. Therefore, ratios such as WBC/PLT, WBC/IL-6, WBC/CRP, and WBC/D-dimer may provide valuable prognostic information by integrating multiple aspects of CCHF pathophysiology.

The insufficient investigation of these parameters in patients diagnosed with CCHF suggests that this study could fill a gap in the literature and potentially provide a new perspective for clinical prognosis evaluation. This study aims to investigate the role of parameters such as leukocyte/platelet, leukocyte/IL-6, leukocyte/CRP, and leukocyte/D-dimer ratios in predicting disease prognosis in patients diagnosed with CCHF.

## 2. Material and Method

### 2.1. Study Population

This retrospective, case–control study was conducted at the Department of Infectious Diseases, Sivas Cumhuriyet University. The sample size was calculated using G*Power version 3.1.9.7 software. To detect a medium effect size (d = 0.65) with a 5% significance level (α), 80% statistical power (1 − β), and a population ratio of 2 (N1/N2), the minimum required sample size was calculated to be 86. The study population consisted of 60 CCHF patients and a control group of 30 individuals. The cases were selected from CCHF patients who were monitored in the Infectious Diseases and Clinical Microbiology Department at Sivas Cumhuriyet University Research Hospital. The diagnosis of CCHF was made using specific real-time polymerase chain reaction test kits for CCHF at the reference laboratory of the General Directorate of Public Health of the Ministry of Health. The control group consisted of 30 apparently healthy individuals whose blood samples were sent to the biochemistry laboratory for various reasons and who had no acute or chronic diseases, normal routine laboratory findings, and no medication use.

### 2.2. Laboratory Analysis

The values of hemoglobin (HGB), PLT, WBC, international normalized ratio (INR), aPTT, D-dimer, fibrinogen, CRP, and IL-6 on the first day of hospital admission were recorded. The results of all these parameters were compared between patient groups requiring intensive care unit (ICU) admission and those who did not, as well as between surviving patients and those with a fatal outcome. Laboratory tests for AST, ALT, GGT, ALP, creatinine and CRP were conducted using photometric methods on a Roche Cobas c702 autoanalyzer (Roche Diagnostics, Germany), while IL-6 levels were assessed using an electrochemiluminescence method on a Roche Cobas e801 autoanalyzer. Complete blood count tests were performed using a Sysmex XN-1000 (Sysmex Corporation, Kobe, Japan) analyzer, and coagulation tests (INR, aPTT, D-dimer, and fibrinogen) were conducted on a Roche Cobas t511 analyzer.

### 2.3. Statistical Analyses

The data collected in this study were analyzed using SPSS software (IBM Corp., SPSS Statistics for Windows, Version 23.0, Armonk, NY, USA). The normality of the data distribution was assessed using the Shapiro–Wilk test. For normally distributed data, the independent *t*-test was employed, while the Mann–Whitney U test was utilized for non-normally distributed data. Categorical variables were analyzed using the Chi-square test or Fisher’s Exact test, as appropriate. Receiver operating characteristic (ROC) analysis was conducted to determine the predictive accuracy and identify cut-off values for diagnosing CCHF, assessing the need for intensive care, and evaluating mortality risk. A significance level of 0.05 was applied.

### 2.4. Ethical Approval

This study was approved by the Sivas Cumhuriyet University Clinical Research Ethics Committee (Date: 21 November 2024, Decision No: 2024-11/28). All participants signed informed consent forms to participate in the study before ethical approval.

## 3. Results

Of the patients, 41 (68.3%) were male, and 19 (31.7%) were female, with an average age of 49.1 ± 15.4 years. In the control group, 21 (70%) were male, with an average age of 47.9 ± 9.3 years. The demographic data of the patients are presented in detail in Table 1. The median and quartiles of the time between the onset of symptoms and admission to hospital were 3 (2–5) days. Laboratory parameters included HGB, PLT, WBC, INR, aPTT, d-dimer, fibrinogen, CRP, and IL-6 values. The results of all these parameters were compared between patients and controls, between those who required intensive care and those who did not, and between fatal cases and survivors. Statistically significant differences were observed in HGB, PLT, WBC, aPTT, INR, fibrinogen, and d-dimer values between the patient and control groups (Table 2).

**Figure 1:**
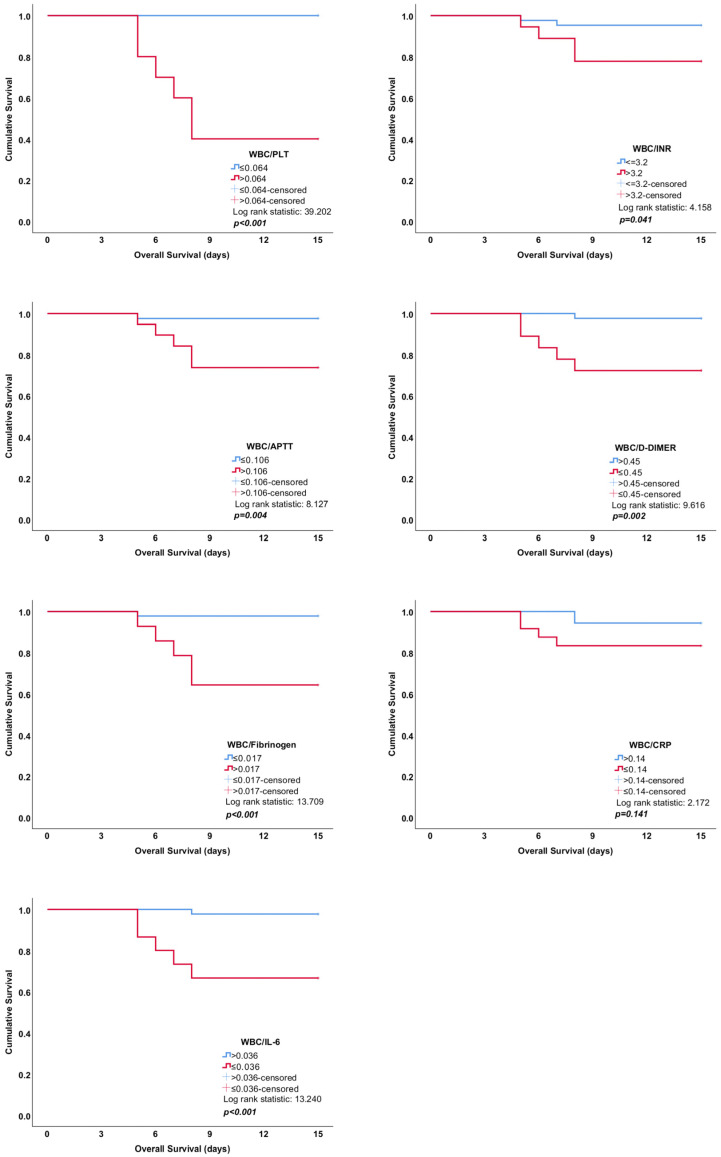
Impact of inflammatory parameters on mortality in CCHF: Kaplan–Meier survival curves.

Statistically significant differences were observed in WBC/aPTT (*p* < 0.001), WBC/fibrinogen (*p* < 0.001), WBC/D-dimer (*p* < 0.001), and WBC/IL-6 (*p* < 0.001) values between the patient and control groups (Table 2). WBC/INR (*p* = 0.04) and WBC/fibrinogen (*p* = 0.002) values were lower in fatal cases compared to survivors. WBC/D-dimer (*p* = 0.002) and WBC/IL-6 (*p* = 0.006) values, on the other hand, were higher in fatal cases compared to survivors (Table 3, Figure 1). In patients requiring ICU, WBC/PLT (*p* < 0.001), WBC/INR (*p* = 0.01), WBC/aPTT (*p* = 0.02), and WBC/fibrinogen (*p* < 0.001) values were higher compared to those not admitted to ICU. However, WBC/D-dimer (*p* = 0.001) and WBC/IL-6 (*p* = 0.006) values were lower in patients requiring ICU compared to those who did not (Table 4). The most promising results based on Kaplan–Meier curves and the log-rank test were identified as WBC/PLT (log rank statistic: 39.202), WBC/Fibrinogen (log rank statistic: 13.709), and WBC/CRP (log rank statistic: 13.240), respectively.

ROC analysis identified WBC/IL-6, WBC/CRP, and WBC/D-dimer as strong diagnostic markers for CCHF, with AUCs of 0.998, 0.980, and 0.990, respectively (Table 5). For predicting mortality, WBC/PLT showed the highest performance (AUC: 0.969), followed by WBC/D-dimer (0.867) and WBC/Fibrinogen (0.880) (Table 6). Similarly, in forecasting ICU need, WBC/PLT again demonstrated excellent accuracy (AUC: 0.984), alongside WBC/Fibrinogen and WBC/D-dimer (AUCs: 0.903 and 0.879, respectively) (Table 7).

## 4. Discussion

Thrombocytopenia is one of the main laboratory parameters of CCHF disease [15]. Lymphocyte count varies according to the immune response of the host. Leucopenia or leukocytosis may be observed [16]. In the present study, WBC and PLT counts were significantly lower in patients compared to controls. However, no difference was found between the patient and control groups in terms of WBC/PLT ratio. This suggests that there was a similar decrease in WBC and PLT levels with no change in the ratio.

Although the factors determining the severity of CCHF have not yet been identified, early detection of disease severity is crucial for reducing mortality rates. Therefore, developing an easy and reliable marker for determining disease severity is very important. In the literature, the prognostic characteristics of inflammatory parameters in CCHF patients have been evaluated. In various studies, low HGB, low PLT, and elevated WBC have been identified as poor prognostic factors [17,18,19,20]. However, some studies reported different results regarding WBC elevation [19,20]. Kazancıoğlu et al. [21] reported that WBC levels did not show differences between fatal cases and survivors. Similarly, Ergonül et al. [22] did not report any difference in WBC levels between fatal cases and survivors. However, both studies found that low PLT levels could be predicting mortality [19,20]. In the CCHF severity scoring system developed by Bakır et al. [8], low PLT and high WBC were included in the scoring system. In the present study, PLT levels were lower, while WBC levels were higher in fatal cases. The WBC/PLT ratio was significantly higher in fatal cases compared to survivors and in patients requiring ICU compared to those who did not. This finding suggests that the WBC/PLT ratio could be a prognostic marker for predicting ICU needs and mortality.

PT and aPTT, which are diagnostic parameters of disseminated intravascular coagulation (DIC), and INR, an important parameter reflecting the functioning of the coagulation cascade, are frequently used coagulation parameters in monitoring CCHF patients and have been associated with poor prognosis [22,23,24]. APTT > 60 s is considered a critical prognostic parameter in CCHF [25,26]. In the present study, consistent with the literature, aPTT and INR values were significantly higher in patients compared to controls, in fatal cases compared to survivors, and in patients requiring intensive care compared to those not requiring intensive care. WBC/aPTT and WBC/INR ratios were significantly lower in patients compared to controls. While WBC/aPTT showed a significant difference between patients requiring ICU and those who did not, no significant difference was observed between fatal cases and survivors. On the other hand, the WBC/INR ratio was significantly higher in fatal cases compared to survivors and in patients requiring ICU compared to those who did not. This finding suggests that the WBC/INR ratio could be a prognostic marker for predicting ICU needs and mortality. However, WBC/aPTT and WBC/INR values were lower in patients compared to controls and higher in fatal cases compared to survivors and in patients requiring intensive care compared to those not requiring intensive care, suggesting that the values in fatal cases and in those requiring ICU were different due to leukocytosis.

The most significant issue during CCHF is bleeding, which frequently manifests as petechiae, ecchymosis, epistaxis, and gum bleeding, while pulmonary and gastrointestinal bleeding can be life-threatening. Bleeding is significantly more common in fatal cases compared to survivors [23]. DIC is a coagulation disorder characterized by widespread intravascular thrombosis and bleeding, and most hemorrhages observed during the hemorrhagic phase are associated with DIC. Excess production of fibrinogen and fibrin degradation leads to the release of fibrin degradation products, further impairing thrombin activation and platelet functions, thereby increasing bleeding diathesis. Fibrinogen and d-dimer are frequently used parameters for diagnosing DIC [25]. Studies evaluating these parameters in CCHF prognosis have shown that D-dimer levels are higher, and fibrinogen levels are lower in fatal cases compared to survivors [20,23,26]. In the present study, consistent with the literature, D-dimer values were higher and fibrinogen values lower in patients compared to controls, and in fatal cases compared to survivors. Supporting this, WBC/D-dimer and WBC/fibrinogen ratios also showed statistically significant differences between patients and control, fatal cases and survivors, as well as between patients requiring ICU and those who did not. This suggests that the WBC/D-dimer and WBC/fibrinogen ratios could be a prognostic marker to predict the need for ICU and mortality.

Endothelial cells and mononuclear cells are the two main targets of the CCHF virus [27,28]. Endothelial damage can occur directly due to the virus’s cytopathic effects or indirectly through chemokines and cytokines released by activated mononuclear cells [27]. Therefore, IL-6, a proinflammatory cytokine, has been frequently studied in CCHF pathogenesis and is significantly higher in fatal cases compared to survivors [9,28,29]. In the present study, consistent with the literature, IL-6 levels were higher in patients compared to controls and in fatal cases compared to survivors. The WBC/IL-6 ratio was also found to be lower in patients compared to controls, in fatal cases compared to survivors, and in those requiring ICU compared to those who did not, with statistically significant differences. This suggests that WBC/IL-6 ratio can be used as a prognostic marker to predict ICU requirement and mortality and as a diagnostic parameter for CCHF.

CRP, an acute phase reactant frequently used in daily practice, is another parameter widely studied in CCHF. While some studies have found CRP to be ineffective in predicting disease progression [30], others have reported significant differences in CRP levels between fatal cases and survivors [26,31]. In the present study, consistent with the literature, CRP levels were significantly higher in patients compared to controls and in fatal cases compared to survivors. There was a difference between patients and controls in terms of WBC/CRP ratio, but this difference was not observed in ICU need and mortality groups. This suggests that the WBC/CRP ratio is not a prognostic marker for predicting the need for ICU or mortality.

In the present study, ROC analysis identified WBC/IL-6, WBC/CRP, and WBC/D-dimer as strong diagnostic markers for CCHF, with AUCs of 0.998, 0.980, and 0.990, respectively.

Current diagnostic tools for CCHF face significant limitations that hinder effective early detection and accessibility in resource-limited settings. Molecular methods like RT-PCR, while highly sensitive and specific for active infections, require sophisticated equipment, trained personnel, and stable laboratory infrastructure, resources often scarce in endemic regions. Antigen detection assays, though simpler, necessitate biosafety level 4 facilities due to the virus’s high pathogenicity, further limiting their use. Serological tests detecting IgM/IgG antibodies are prone to delayed utility, as antibodies emerge days after symptom onset and may be undetectable in severe cases with compromised immune responses. Additionally, virus neutralization assays, though valuable for research, lack diagnostic practicality due to low neutralizing antibody levels in infections. Commercial tests remain unaffordable or inaccessible in many endemic areas due to geopolitical instability and financial constraints. Collectively, these challenges underscore the urgent need for rapid, affordable, and field-deployable diagnostics to improve early case management and outbreak control [32,33,34]. Accordingly, ratios of inflammatory markers which have exhibited robust diagnostic performance hold significant promise as cost-effective and rapidly deployable tools for early CCHF detection in resource-limited settings.

While traditional severity scoring systems such as the CCHF SGS have been used to predict outcomes, they require multiple clinical and laboratory parameters [8]. In contrast, the inflammatory marker ratios evaluated in this study are easily obtainable from routine laboratory tests and may provide a simpler yet effective tool for risk stratification. However, further prospective studies are needed to validate these findings and assess their integration into clinical practice. A major strength of our study is the focus on inflammatory marker ratios rather than individual biomarkers, which may better capture the dynamic nature of the host response to viral infection.

This study has several limitations. Its retrospective, single-center design introduces potential selection bias and restricts generalizability, necessitating prospective validation in diverse cohorts to confirm the prognostic utility of identified markers. The timing of laboratory measurements relative to symptom onset was not standardized, which may affect the interpretation of dynamic biomarkers like platelet counts or liver enzymes. Additionally, the inclusion of patients at varying disease stages without systematic criteria for defining illness progression limits our ability to assess how early prognostic markers correlate with outcomes. Future work should prioritize prospective designs with serial biomarker assessments and external validation.

## 5. Conclusions

This study highlights the potential prognostic value of inflammatory and coagulation marker ratios, such as WBC/PLT, WBC/INR, WBC/D-dimer, and WBC/IL-6, in predicting disease severity, ICU admission, and mortality risk. The findings suggest that these ratios could serve as useful biomarkers for early risk stratification and clinical decision-making in CCHF patients, particularly indicating that WBC/PLT and WBC/fibrinogen are distinctive in predicting mortality. Unlike traditional severity scoring systems, these parameters are easily obtainable from routine laboratory tests, making them practical for bedside application.

## Figures and Tables

**Table 1:** Demographic characteristics of the patients.

Variables	Patients (n = 60)	Controls (n = 30)	*p*-Value
Age (Years) ± SD	49.1 ± 15.4	47.9 ± 9.3	0.648
Gender			0.872
Male, n (%)	41 (68.3)	21 (70)	
Female, n (%)	19 (31.7)	9 (30)	
Comorbidities			
Hypertension	12 (20.0)		
Diabetes mellitus	6 (10.0)		
Coronary artery disease	4 (6.7)		
Chronic obstructive lung disease	7 (11.7)		
Malignancy	2 (3.3)		
Laboratory findings			
HGB (g/dL)	13.8 ± 1.6	14.7 ± 1.6	**0.009**
PLT (10^9^/L)	82 (53–112)	247 (192–272)	**<0.001**
WBC (10^9^/L)	2.59 (1.6–4.28)	7.0 (5.9–9.0)	**<0.001**
INR	1.0 (0.9–1.2)	1.0 (1.0–1.10)	0.382
aPTT (s)	32 (29–35)	29 (26–31)	0.002
D-dimer (mg/L FEU)	2.2 (0.9–7.03)	0.2 (0.2–0.2)	**<0.001**
Fibrinogen (mg/dL)	268 ± 50	270 ± 56	0.846
AST (U/L)	88 (46–191)	17 (15–20)	**<0.001**
ALT (U/L)	53 (26–113)	18 (13–25)	**<0.001**
GGT (U/L)	45 (23–95)	19 (13–29)	**<0.001**
ALP (U/L)	75 (60–94)	67 (60–79)	0.139
Creatinine (mg/dL)	0.8 (0.7–1.0)	0.8 (0.7–0.9)	0.196
CRP (mg/L)	12.0 (4.0–35)	0.95 (0.41–2.16)	**<0.001**
IL-6 (ng/L)	22 (13–76)	1.5 (1.5–1.99)	**<0.001**
Treatment, n (%)			
Ribavirin	6 (10.0)		
Steroid	22 (36.7)		
Thrombocytes	28 (46.7)		
Fresh frozen plasma	41 (68.3)		
Antibiotics	20 (33.3)		
ICU need, n (%)	7 (11.7)		
Mortality, n (%)	6 (10.0)		

Age, hemoglobin and fibrinogen values are given as mean ± SD. Other laboratory parameters are given as median and quartiles (Q1–Q3). Categorical data were analyzed using the Chi-square test, while continuous variables were compared using either the Mann–Whitney U test or the independent sample *t*-test, depending on the data distribution. Bold values indicate statistical significance (*p* < 0.05). SD, Standard deviation; HGB, hemoglobin; PLT, platelet; WBC, white blood cell; INR, international normalized ratio; aPTT, activated partial thromboplastin time; AST, aspartate aminotransferase; ALT, alanine aminotransferase; GGT, gama-glutamyl transferase; ALP, alkaline phosphates; CRP, C-reactive protein; IL-6, interleukin 6; ICU, intensive care unit.

**Table 2:** Comparison of inflammatory parameter ratios of patient and control groups.

Variables	Patients (n = 60)	Control (n = 30)	*p*-Value
WBC/PLT	0.03 (0.02–0.05)	0.03 (0.02–0.04)	0.909
WBC/INR	2.33 (1.65–3.74)	7.48 (5.9–8.9)	**<0.001**
WBC/aPTT	0.08 (0.05–0.12)	0.26 (0.22–0.33)	**<0.001**
WBC/D-dimer	0.96 (0.36–2.53)	35 (29.5–45)	**<0.001**
WBC/Fibrinogen	0.01 (0.01–0.02)	0.03 (0.02–0.04)	**<0.001**
WBC/CRP	0.19 (0.08–0.55)	6.81 (3.35–17.5)	**<0.001**
WBC/IL-6	0.11 (0.04–0.18)	3.97 (3.38–5.67)	**<0.001**

Laboratory parameters are given as median and quartiles (Q1–Q3). Variables were compared using the Mann–Whitney U test. Bold values indicate statistical significance (*p* < 0.05). WBC, white blood cells; PLT, platelets; INR, international normalized ratio; aPTT, activated partial thromboplastin time; CRP, C-reactive protein; IL-6, interleukin-6.

**Table 3:** Comparison of inflammatory parameters in fatal cases and survivors.

Laboratory Finding	Non-Fatal Cases (n = 54)	Fatal Cases (n = 6)	*p*-Value
HGB (g/dL)	13.94 ± 1.51	12.22 ± 1.79	**0.012**
PLT (10^9^/L)	85 (57–115)	38 (31–51)	**0.005**
WBC (10^9^/L)	2.4 (1.6–3.5)	6.8 (4.9–9.8)	**0.010**
INR	1.0 (0.9–1.1)	1.5 (1.5–1.6)	**<0.001**
aPTT (s)	32 (28–35)	45 (42–64)	**<0.001**
D-dimer (mg/L FEU)	1.85 (0.81–6.0)	38.0 (15.0–45.0)	**<0.001**
Fibrinogen (mg/dL)	273 ± 48	226 ± 61	**0.029**
CRP (mg/L)	10.0 (4.0–26.0)	101 (35–166)	**0.001**
IL-6 (ng/L)	18.5 (12.0–53.0)	314 (79–585)	**<0.001**
WBC/PLT	0.03 (0.02–0.04)	0.15 (0.1–0.32)	**<0.001**
WBC/INR	2.33 (1.6–3.38)	5.22 (3.27–6.53)	**0.043**
WBC/aPTT	0.08 (0.05–0.11)	0.16 (0.11–0.22)	0.074
WBC/D-dimer	1.13 (0.5–3.0)	0.17 (0.11–0.4)	**0.002**
WBC/Fibrinogen	0.01 (0.01–0.01)	0.03 (0.02–0.06)	**0.002**
WBC/CRP	0.21 (0.08–0.63)	0.1 (0.03–0.14)	0.078
WBC/IL-6	0.12 (0.04–0.21)	0.02 (0.02–0.04)	**0.006**

Hemoglobin and fibrinogen values are given as mean ± SD. Other laboratory parameters are given as median and quartiles (Q1–Q3). Variables were compared using either the Mann–Whitney U test or the independent sample *t*-test, depending on the data distribution. Bold values indicate statistical significance (*p* < 0.05). HGB, Hemoglobin; PLT, platelet; WBC, white blood cell; INR, international normalized ratio; aPTT, activated partial thromboplastin time; CRP, C-reactive protein; IL-6, interleukin-6.

**Table 4:** Comparison of the inflammatory parameters of patients who needed ICU and those who did not.

Variables	ICU Requirement	No Need for ICU	*p*-Value
WBC/PLT	0.16 (0.1–0.32)	0.03 (0.02–0.04)	**<0.001**
WBC/INR	6.18 (3.27–6.53)	2.33 (1.6–3.2)	**0.013**
WBC/aPTT	0.19 (0.11–0.22)	0.08 (0.05–0.11)	**0.026**
WBC/D-dimer	0.15 (0.11–0.4)	1.18 (0.51–3)	**0.001**
WBC/Fibrinogen	0.03 (0.02–0.06)	0.01 (0.01–0.01)	**<0.001**
WBC/CRP	0.09 (0.03–0.14)	0.21 (0.08–0.63)	0.061
WBC/IL-6	0.02 (0.02–0.05)	0.12 (0.04–0.21)	**0.006**

Laboratory parameters are given as median and quartiles (Q1–Q3). Variables were compared using the Mann–Whitney U test. Bold values indicate statistical significance (*p* < 0.05). WBC, White blood cell; PLT, platelet; INR, international normalized ratio; aPTT, activated partial thromboplastin time; CRP, C-reactive protein; IL-6, interleukin-6.

**Table 5:** ROC analysis results for predicting the CCHF diagnosis.

Variables	Cut-Off Value	AUC	Sensitivity (95% CI)	Specificity (95% CI)	LR (+) (95% CI)	LR (−) (95% CI)	*p*-Value
WBC/PLT	≤0.045	0.512	71.7 (58.6–82.5)	0 (0–11.6)	0.72 (0.61–0.84)	NA	0.835
WBC/INR	≤3.5	0.923	73.3 (60.3–83.9)	100 (88.4–100)	NA	0.27 (0.18–0.41)	**<0.001**
WBC/APTT	≤0.12	0.917	78.3 (65.8–87.9)	96.7 (82.8–99.9)	23.5 (3.41–162)	0.22 (0.14–0.36)	**<0.001**
WBC/D-dimer	≤11.1	0.990	98.3 (91.1–100)	100 (88.4–100)	NA	0.017 (0.002–0.12)	**<0.001**
WBC/Fibrinogen	≤0.017	0.886	81.7 (69.6–90.5)	90.0 (73.5–97.9)	8.17 (2.77–24.1)	0.20 (0.12–0.35)	**<0.001**
WBC/CRP	≤2.1	0.980	95.0 (86.1–99.0)	96.7 (82.8–99.9)	28.5 (4.15–196)	0.052 (0.017–0.16)	**<0.001**
WBC/IL-6	≤0.53	0.998	96.7 (88.5–99.6)	100 (88.4–100)	NA	0.033 (0.009–0.13)	**<0.001**

Bold values indicate statistical significance (*p* < 0.05). AUC, Area Under Curve; LR, likelihood ratio; CI, confidence interval; NA, not applicable; WBC, white blood cell; PLT, platelet; INR, international normalized ratio; aPTT, activated partial thromboplastin time; CRP, C-reactive protein; IL-6, interleukin-6.

**Table 6:** ROC analysis results for predicting the mortality risk of CCHF patients.

Variables	Cut-Off Value	AUC	Sensitivity (95% CI)	Specificity (95% CI)	LR (+) (95% CI)	LR (−) (95% CI)	*p*-Value
WBC/PLT	>0.064	0.969	100 (54.1–100)	92.6 (82.1–97.9)	13.5 (5.26–34.7)	0	**<0.001**
WBC/INR	>3.2	0.753	83.3 (35.9–99.6)	74.1 (60.3–85.0)	3.21 (1.81–5.72)	0.23 (0.037–1.36)	0.061
WBC/APTT	>0.106	0.722	83.3 (35.9–99.6)	74.1 (60.3–85.0)	3.21 (1.81–5.72)	0.23 (0.037–1.36)	0.096
WBC/D-dimer	≤0.45	0.867	100 (54.1–100)	75.9 (62.4–86.5)	4.15 (2.59–6.67)	0	**<0.001**
WBC/Fibrinogen	>0.017	0.880	83.3 (35.9–99.6)	88.9 (77.4–95.8)	7.5 (3.25–17.3)	0.19 (0.031–1.12)	**<0.001**
WBC/CRP	≤0.14	0.722	83.3 (35.9–99.6)	63.0 (48.7–75.7)	2.25 (1.37–3.71)	0.26 (0.044–1.60)	**0.028**
WBC/IL-6	≤0.036	0.830	83.3 (35.9–99.6)	81.5 (68.6–90.7)	4.5 (2.32–8.74)	0.2 (0.034–1.23)	**<0.001**

Bold values indicate statistical significance (*p* < 0.05). AUC, Area Under Curve; LR, likelihood ratio; CI, confidence interval; NA, not applicable; WBC, white blood cell; PLT, platelet; INR, international normalized ratio; aPTT, activated partial thromboplastin time; CRP, C-reactive protein; IL-6, interleukin-6.

**Table 7:** ROC analysis results for predicting the intensive care requirements of CCHF patients.

Variables	Cut-Off Value	AUC	Sensitivity (95% CI)	Specificity (95% CI)	LR (+) (95% CI)	LR (−) (95% CI)	*p*-Value
WBC/PLT	>0.064	0.984	100 (59.0–100)	94.3 (84.3–98.8)	17.7 (5.89–53.0)	0	**<0.001**
WBC/INR	>3.2	0.784	85.7 (42.1–99.6)	75.5 (61.7–86.2)	3.49 (1.99–6.12)	0.19 (0.031–1.17)	**0.017**
WBC/APTT	>0.106	0.757	85.7 (42.1–99.6)	75.5 (61.7–86.2)	3.49 (1.99–6.12)	0.19 (0.031–1.17)	**0.030**
WBC/D-dimer	≤0.45	0.879	100 (59.0–100)	77.4 (63.8–87.7)	4.42 (2.69–7.26)	0	**<0.001**
WBC/Fibrinogen	>0.017	0.903	85.7 (42.1–99.6)	90.6 (79.3–96.9)	9.09 (3.74 22.1)	0.16 (0.026–0.97)	**<0.001**
WBC/CRP	≤0.14	0.720	85.7 (42.1–99.6)	64.2 (49.8–76.9)	2.39 (1.49–3.83)	0.22 (0.036–1.38)	**0.014**
WBC/IL-6	≤0.048	0.814	85.7 (42.1–99.6)	71.7 (57.7–83.2)	3.03 (1.79–5.12)	0.2 (0.032–1.23)	**<0.001**

Bold values indicate statistical significance (*p* < 0.05). AUC, Area Under Curve; LR, likelihood ratio; CI, confidence interval; NA, not applicable; WBC, white blood cell; PLT, platelet; INR, international normalized ratio; aPTT, activated partial thromboplastin time; CRP, C-reactive protein; IL-6, interleukin-6.

## Data Availability

The original contributions presented in this study are included in the article. Further inquiries can be directed to the corresponding author(s).
